# Supplementary material for: Prevalence and Genomic Diversity of Salmonella enterica Recovered from River Water in a Major Agricultural Region in Northwestern Mexico
Source: Microorganisms. 2022 Jun 14;10(6):1214. doi: 10.3390/microorganisms10061214 (PMC9228531; doi:10.3390/microorganisms10061214)
Supplement: Supplementary file 1 [file microorganisms-10-01214-s001.zip › Supplementary Table S1_Genome characteristics of the Salmonella enterica isolates.pdf]

Supplementary Table S1. Genome characteristics of *Salmonella enterica* strains isolates from river waters.

Bacterial Isolate	Sampling date	Sampling site	Sampling river	*S. enterica* Serovar	Sequence Type	Sequencing platform	Contigs	Genome size	Longest contigs	N50 [a]	L50 [b]	% GC	Coverage	Genome accession
IRV-1	06/2018	A	Humaya	Minnesota	548	Miseq	36	4562051	659242	251865	6	52.18	148	JAMBXM000000000
IRV-2	06/2018	B	Humaya	Saintpaul	50	Miseq	29	4698291	1334712	580937	3	52.23	163	JAMBXL000000000
IRV-3	06/2018	E	Tamazula	Braenderup	22	Miseq	44	4669019	1120534	233050	4	52.26	154.5	JAMBXK000000000
IRV-5	06/2018	G	Culiacan	Braenderup	22	Miseq	66	4665579	528067	182531	9	52.25	151	JAMBXJ000000000
IRV-11	06/2018	A	Humaya	Newport	45	Miseq	47	4687539	656971	225565	7	52.2	192.4	JAMBXI000000000
IRV-12	06/2018	B	Humaya	Saintpaul	50	Miseq	43	4741533	858815	225894	6	52.2	179.9	JAMBXH000000000
IRV-14	06/2018	D	Tamazula	Oranienburg	23	Miniseq	261	4578525	130638	34317	38	52.18	64.8	JAMBXG000000000
IRV-15	06/2018	E	Tamazula	Oranienburg	23	Miseq	121	4991623	331131	105828	13	51.96	160.5	JAMBXF000000000
IRV-16	06/2018	F	Tamazula	Montevideo	81	Miseq	63	4614272	835924	196368	6	52.2	161.3	JAMBXE000000000
IRV-17	06/2018	G	Culiacan	Give	654	Miseq	51	4970520	909760	334064	5	52.07	134.4	JAMBXD000000000
IRV-18	06/2018	H	Culiacan	Sandiego	3541	Miseq	55	4784818	456173	248949	8	51.96	198.8	JAMBXC000000000
IRV-19	06/2018	I	Culiacan	Oranienburg	23	Miseq	30	4678332	823478	325049	5	52.08	172.2	JAMBXB000000000
IRV-21	06/2018	J	Culiacan	Oranienburg	23	Miniseq	297	4665423	127162	33227	44	52.2	69.2	JAMBXA000000000
IRV-23	06/2018	K	Culiacan	Oranienburg	23	Miseq	43	4612312	543057	225428	7	52.06	159.9	JAMBWZ000000000
IRV-24	07/2018	A	Humaya	Montevideo	81	Miseq	49	4629430	820852	207857	6	52.24	180.3	JAMBWY000000000
IRV-25	07/2018	B	Humaya	Oranienburg	23	Miseq	35	4613527	707199	271315	5	52.08	62	JAMBWX000000000
IRV-26	07/2018	C	Humaya	Anatum	26	Miseq	38	4687212	671644	289977	5	52.16	147.3	JAMBWW000000000
IRV-27	07/2018	D	Tamazula	Bovismorbificans	150	Miseq	64	5089867	508487	252177	8	52.07	200.3	JAMBWV000000000
IRV-28	07/2018	E	Tamazula	Anatum	64	Miseq	26	4672342	912431	431865	4	52.17	156.8	JAMBWU000000000
IRV-29	07/2018	F	Tamazula	Oranienburg	23	Miseq	31	4578301	782334	345819	4	52.11	149.8	JAMBWT000000000

IRV-30	07/2018	G	Culiacan	Meleagridis	463	Miseq	86	4869077	655520	295423	6	51.99	126.1	JAMBWS000000000
IRV-31	07/2018	H	Culiacan	Typhimurium	19	Miseq	89	4895948	623165	139289	10	52.16	115.3	JAMBWR000000000
IRV-32	07/2018	I	Culiacan	Oranienburg	23	Miseq	40	4614328	706873	214894	7	52.08	143.7	JAMBWQ000000000
IRV-33	07/2018	J	Culiacan	Pomona	451	Miseq	82	5071795	692829	249558	6	52.17	159.3	JAMBWP000000000
IRV-34	07/2018	D	Tamazula	Sandiego	3541	Miseq	70	4841814	355995	158982	11	52.06	142.3	JAMBWO000000000
IRV-35	08/2018	A	Humaya	Montevideo	81	Miseq	35	4628440	910412	718108	3	52.27	167.3	JAMBWN000000000
IRV-36	08/2018	B	Humaya	Anatum	64	Miseq	27	4839777	1333852	347797	4	52.05	135.9	JAMBWM000000000
IRV-37	08/2018	C	Humaya	Anatum	64	Miseq	23	4654318	1649524	679504	2	52.08	351	JAMBWL000000000
IRV-38	08/2018	D	Tamazula	Oranienburg	23	Miseq	33	4616903	1332877	471408	3	52.08	172.8	JAMBWK000000000
IRV-40	08/2018	D	Tamazula	Gaminara	239	Miseq	33	4611090	659813	259412	5	52.24	172.2	JAMBWJ000000000
IRV-41	08/2018	F	Tamazula	Oranienburg	23	Miniseq	451	4603880	204519	20666	64	52.33	52.2	JAMBWI000000000
IRV-42	08/2018	G	Culiacan	Agona	13	Miniseq	555	4724968	82584	17482	83	52.25	43.8	JAMBWH000000000
IRV-43	08/2018	H	Culiacan	Weltevreden	365	Miseq	102	5068775	353989	114039	16	52.01	169.9	JAMBWG000000000
IRV-44	08/2018	I	Culiacan	Pomona	451	Miseq	60	4906265	658825	317986	5	52.06	163.4	JAMBWF000000000
IRV-46	12/2018	D	Tamazula	Muenchen	112	Miseq	67	5165337	775711	220662	7	51.98	182.8	JAMBWE000000000
IRV-47	12/2018	F	Tamazula	Montevideo	81	Miseq	33	4654112	738382	393395	4	52.3	176.9	JAMBWD000000000
IRV-48	12/2018	H	Culiacan	Oranienburg	23	Miseq	31	4719336	593775	386077	5	52.06	154.6	JAMBWC000000000
IRV-49	12/2018	I	Culiacan	Pomona	451	Miseq	57	4907888	721118	540826	4	52.05	158.8	JAMBWB000000000
IRV-50	01/2019	G	Culiacan	Bovismorbificans	150	Miseq	48	4608871	722390	210468	7	52.16	169.9	JAMBWA000000000
IRV-51	04/2019	A	Humaya	Give	654	Miseq	85	4976688	546080	316450	6	52.05	167.8	JAMBVZ000000000
IRV-52	02/2019	C	Humaya	Sandiego	3541	Miseq	71	4904592	357025	162394	10	52.04	149.7	JAMBVY000000000
IRV-54	03/2019	B	Humaya	Weltevreden	365	Miseq	74	5012123	354597	144269	12	52.1	159.4	JAMBVX000000000

IRV-55	03/2019	B	Humaya	Weltevreden	365	Miseq	86	5013520	354625	127209	13	52.1	181.2	JAMBVW000000000
IRV-56	03/2019	F	Tamazula	Poona	447	Miniseq	418	4939735	207439	25101	59	52.13	38.5	JAMBVV000000000
IRV-57	04/2019	A	Humaya	Oranienburg	23	Miniseq	675	4592386	73666	13436	97	52.49	23	JAMBVU000000000
IRV-58	04/2019	C	Humaya	Oranienburg	23	Miniseq	340	4697264	99711	30387	48	52.25	64.4	JAMBVT000000000
IRV-59	04/2019	E	Tamazula	Soahanina	970	Miseq	86	4971302	630389	137219	11	52.16	161.8	JAMBVS000000000
IRV-60	04/2019	I	Culiacan	Soahanina	970	Miseq	92	5109402	584982	143554	11	52.03	144.4	JAMBVR000000000
IRV-61	07/2019	B	Humaya	Anatum	64	Miniseq	344	4593609	121303	27340	50	52.31	58.9	JAMBVQ000000000
IRV-62	07/2019	C	Humaya	Oranienburg	23	Miniseq	282	4600651	229487	32537	40	52.23	56.6	JAMBVP000000000
IRV-63	07/2019	D	Tamazula	Soahanina	970	Miniseq	469	4824586	130317	19791	72	52.35	47.1	JAMBVO000000000
IRV-64	07/2019	E	Tamazula	Sandiego	3541	Miniseq	615	4914227	128799	19953	67	52.22	47	JAMBVN000000000
IRV-65	07/2019	F	Tamazula	Infantis	32	Miniseq	324	4567957	90170	26214	59	52.39	54.8	JAMBVM000000000
IRV-66	07/2019	G	Culiacan	Anatum	64	Miniseq	263	4557610	110547	36230	41	52.3	64.8	JAMBVL000000000
IRV-67	07/2019	H	Culiacan	Pomona	451	Miniseq	840	4920645	53237	11391	128	52.39	38.5	JAMBVK000000000
IRV-69	07/2019	K	Culiacan	Saintpaul	50	Miniseq	325	4692411	108100	28015	52	52.35	57.7	JAMBVJ000000000
IRV-70	08/2019	A	Humaya	Minnesota	285	Miniseq	546	4899967	80511	17283	85	52.43	52.8	JAMBVI000000000
IRV-71	08/2019	B	Humaya	Saintpaul	50	Miniseq	613	4607026	56578	14735	99	52.54	28.7	JAMBVH000000000
IRV-72	08/2019	D	Tamazula	Saintpaul	50	Miniseq	186	4671484	250025	50192	27	52.29	80.2	JAMBVG000000000
IRV-74	08/2019	F	Tamazula	Give	654	Miniseq	696	5493370	110317	23416	72	52.25	47.5	JAMBVF000000000
IRV-75	08/2019	I	Culiacan	Oranienburg	23	Miniseq	140	4742492	473461	83581	19	52.08	92.1	JAMBVE000000000
IRV-76	08/2019	J	Culiacan	Javiana	24	Miniseq	354	4567489	91493	26681	53	52.28	50.6	JAMBVD00000000
IRV-77	08/2019	K	Culiacan	Carrau	226	Miniseq	358	4807635	108929	24828	56	52.34	58.3	JAMBVC000000000
IRV-78	10/2019	A	Humaya	Give	654	Miseq	77	4981785	909132	316460	5	52.06	131.4	JAMBVB000000000

IRV-79	10/2019	B	Humaya	Oranienburg	23	Miniseq	389	4579796	74621	21553	68	52.32	61.8	JAMBVA000000000
IRV-80	10/2019	C	Humaya	Albany	292	Miniseq	395	4742206	115558	22382	60	52.27	43.5	JAMBUZ000000000
IRV-81	10/2019	E	Tamazula	Abaetetuba	2041	Miniseq	533	4935788	88314	18897	77	52.33	38.9	JAMBUY000000000
IRV-82	10/2019	H	Culiacan	Senftenberg	14	Miniseq	608	4807472	94042	15265	94	52.35	49.3	JAMBUX000000000
IRV-83	10/2019	I	Culiacan	Oranienburg	23	Miniseq	492	4619687	110643	18343	73	52.28	39.9	JAMBUW000000000
IRV-84	11/2019	C	Humaya	Yoruba	1316	Miniseq	599	4663594	96873	15146	89	52.39	20.6	JAMBUV000000000
IRV-85	11/2019	D	Tamazula	Saphra	2310	Miniseq	349	4607634	111971	24221	54	52.38	49.5	JAMBUU000000000
IRV-86	11/2019	E	Tamazula	Typhimurium	19	Miniseq	412	4834449	138760	24380	64	52.34	53.5	JAMBUT000000000
IRV-87	11/2019	G	Culiacan	Javiana	24	Miniseq	415	4586256	166990	27822	50	52.38	57.6	JAMBUS000000000

[a] N50. Length of the contigs, such that using contigs of equal or greater size produces half the bases of the genome

[b] L50. Count of the smallest number of contigs whose sum in length constitutes half the size of the genome
