# Supplementary material for: Prevalence and Genomic Diversity of Salmonella enterica Recovered from River Water in a Major Agricultural Region in Northwestern Mexico
Source: Microorganisms. 2022 Jun 14;10(6):1214. doi: 10.3390/microorganisms10061214 (PMC9228531; doi:10.3390/microorganisms10061214)
Supplement: Supplementary file 1 [file microorganisms-10-01214-s001.zip › Supplementary Table S2_Nonsynonymous SNPs identified in S. enterica isolates.pdf]

Supplementary Table S2. List of nonsynonymous single nucleotide polymorphisms identified in the sequenced *Salmonella enterica* isolates recovered from river water in the Culiacan Valley.

Locus tag	Position in Reference *S.* Typhimurium strain LT2	Nucleotide difference in clade 1 isolates	Nucleotide difference in clade 2 isolates	Amino acid difference in clade 1 isolates	Amino acid difference in clade 2 isolates	Gene name	Description of gene product	Functional category
STM0177	208,411	C	G	Serine	Threonine	*stiA*	Starvation-inducible loci required for survival during prolonged nutrient deprivation.	Virulence
STM0177	208,498	T	G	Glutamate	Alanine	*stiA*	Starvation-inducible loci required for survival during prolonged nutrient deprivation.	Virulence
STM0177	208,695	A	T	Aspartate	Glutamate	*stiA*	Starvation-inducible loci required for survival during prolonged nutrient deprivation.	Virulence
STM0177	208,811	G	T	Glutamine	Lysine	*stiA*	Starvation-inducible loci required for survival during prolonged nutrient deprivation.	Virulence
STM0177	208,898	C	T	Alanine	Threonine	*stiA*	Starvation-inducible loci required for survival during prolonged nutrient deprivation.	Virulence
STM0180	212,207	A	T	Phenylalanine	Isoleucine	*panC*	Predicted protein involved in pantothenate (water-soluble vitamin) biosynthesis.	Metabolism
STM0183	213,796	T	C	Asparagine	Aspartate	*folK*	Hydroxymethyldihydropterin pyrophosphokinase required for folate biosynthesis.	Metabolism
STM0185	215,489	C	T	Alanine	Threonine	*yadB*	tRNA modifying enzyme involved in the general stress response.	Metabolism
STM0194	229,134	T	A	Leucine	Isoleucine	*fhuB*	Siderophore transport protein from the periplasm to the cytoplasm.	Metabolism
STM0220	258,717	G	A	Alanine	Threonine	*dxr*	Reductoisomerase responsible for generating isoprenoid precursors.	Metabolism
STM0373	427,824	A	C	Isoleucine	Leucine	*yaiU*	Putative protein involved in flagellar movement in response to chemoreceptor signals.	Motility

STM0381	434,974	A	G	Threonine	Alanine	STM0381	Putative inner membrane protein.	Membrane
STM0497	557,371	C	G	Glutamate	Glutamine	STM0497	Putative periplasmic protein.	Membrane
STM0595	657,313	A	C	Glutamine	Histidine	*entC*	Isochorismate synthase for the biosynthesis of the iron-chelating agent enterobactin.	Metabolism
STM0800	868,768	A	G	Isoleucine	Valine	*slrP*	Ubiquitin ligase targeting mammalian thioredoxin with a role in host cell apoptosis.	Virulence
STM0800	868,891	T	A	Leucine	Isoleucine	*slrP*	Ubiquitin ligase targeting mammalian thioredoxin with a role in host cell apoptosis.	Virulence
STM0884	954,886	A	T	Isoleucine	Leucine	STM0884	Putative inner membrane protein co-regulated with the SPI-2 type III secretion system	Virulence
STM1064	1,156,242	C	G	Alanine	Glycine	*pqiB*	Paraquat-inducible protein B upregulated in response to oxidative stress.	Virulence
STM1070	1,161,603	C	G	Alanine	Proline	*ompA*	Outer membrane protein A involved in many pathogenesis-related processes	Virulence
STM1143	1,231,550	G	T	Glutamine	Histidine	*csgB*	Minor curlin subunit involved in curli and cellulose biosynthesis.	Virulence
STM1392	1,479,235	T	G	Isoleucine	Leucine	*ssrA*	Secretion system sensor kinase with a role in virulence and response to the environment.	Virulence
STM1416	1,497,115	A	T	Threonine	Serine	*ssaO*	Secretion system apparatus protein in *Salmonella* pathogenicity island 2.	Virulence
STM1490	1,567,963	A	C	Serine	Alanine	STM1490	Putative chloride channel membrane protein.	Membrane
STM1540	1,615,434	G	A	Valine	Isoleucine	STM1540	Putative secreted hydrolase.	Virulence
STM1611	1,701,535	T	C	Glutamine	Arginine	*rimL*	Acetyl transferase responsible for N(alpha)-acetylation of ribosomal proteins.	Metabolism
STM1637	1,728,271	C	T	Histidine	Tyrosine	STM1637	Putative ABC transporter, ATP-binding protein	Membrane
STM1667	1,760,100	T	C	Lysine	Glutamate	STM1667	Putative thiol peroxidase.	Virulence
STM1670	1,763,646	A	C	Serine	Alanine	STM1670	Putative serine/threonine protein kinase.	Metabolism
STM1703	1,796,570	G	A	Serine	Asparagine	*yciR*	Regulator of cellulose production and biofilm formation	Virulence
STM1713	1,808,805	T	G	Lysine	Glutamine	*cysB*	Transcriptional activator LysR family regulating genes of the cysteine regulon.	Transcription
STM1790	1,889,160	T	G	Leucine	Arginine	STM1790	Putative thiol-disulfide isomerase and thioredoxin	Metabolism

STM1931	2,026,137	A	G	Serine	Proline	*araH*	Putative intracellular protease/amidase with a role in virulence	Virulence
STM2016	2,098,184	T	C	Methionine	Valine	*cobT*	Nicotinate mononucleotide with a key role in synthesis of cobalamin, a nutrient in the vitamin B complex.	Metabolism
STM2016	2,098,250	C	T	Alanine	Threonine	*cobT*	Nicotinate mononucleotide with a key role in synthesis of cobalamin, a nutrient in the vitamin B complex.	Metabolism
STM2301	2,410,321	T	C	Valine	Alanine	*pqaB*	Lipid transferase associated with antimicrobial resistance and host invasion.	Virulence
STM2428	2,541,842	G	A	Proline	Serine	*zipA*	Inner membrane protein required for the septal ring structure that mediates cell division.	Membrane
STM2503	2,620,423	C	T	Methionine	Isoleucine	STM2503	Putative diguanylate cyclase with a posivite effect in motility.	Motility
STM2515	2,645,063	G	C	Leucine	Valine	*ratA*	Outer membrane protein on Salmonella pathogeneicity island CS54 required for host colonization.	Virulence
STM2515	2,646,218	T	C	Threonine	Alanine	*ratA*	Outer membrane protein on Salmonella pathogeneicity island CS54 required for host colonization.	Virulence
STM2515	2,647,139	A	T	Serine	Threonine	*ratA*	Outer membrane protein on Salmonella pathogeneicity island CS54 required for host colonization.	Virulence
STM2515	2,647,150	G	C	Threonine	Serine	*ratA*	Outer membrane protein on Salmonella pathogeneicity island CS54 required for host colonization.	Virulence
STM2517	2,649,769	C	T	Valine	Isoleucine	*sinH*	Invasion protein on Salmonella pathogeneicity island CS54 required for host colonization.	Virulence
STM2517	2,650,327	C	A	Valine	Phenylalanine	*sinH*	Invasion protein on Salmonella pathogeneicity island CS54 required for host colonization.	Virulence
STM2517	2,650,636	T	A	Threonine	Serine	*sinH*	Invasion protein on Salmonella pathogeneicity island CS54 required for host colonization.	Virulence

STM2891	3,034,506	T	C	Threonine	Alanine	*spaO*	Factor required for sorting platform for effectors to be secreted through the type III secretion system.	Virulence
STM2898	3,042,269	C	A	Alanine	Serine	*invG*	Outer membrane secretion channel for the effector proteins of type III secretion system.	Virulence
STM2961	3,112,004	G	T	Threonine	Lysine	*ygcY*	Putative glucarate dehydratase	Metabolism
STM3173	3,336,451	A	C	Serine	Alanine	*plsC*	Acyltransferase required for the synthesis of membrane of phospholipids.	Membrane
STM3478	3,630,670	C	T	Alanine	Valine	*bigA*	Putative surface-exposed virulence protein.	Virulence
STM3488	3,644,428	A	G	Valine	Alanine	*hofQ*	Putative outer membrane DNA translocase associated with type II secretion system.	Virulence
STM3490	3,644,801	T	C	Isoleucine	Methionine	*yrfB*	Putative inner membrane protein.	Membrane
STM3545	3,714,851	G	C	Glutamine	Glutamate	*yhhX*	Putative oxidoreductase.	Metabolism
STM3693	3,888,211	C	G	Aspartate	Glutamate	*lldR*	Positive transcription regulator of lactate utilization.	Transcription
STM3757	3,955,675	T	G	Aspartate	Glutamate	*misL*	Autotransporter protein required for adhesion and intestinal colonization in the host.	Virulence
STM3810	4,010,667	T	G	Leucine	Tryptophan	*yidQ*	Putative outer membrane lipoprotein.	Membrane
STM4049	4,260,637	C	T	Arginine	Cysteine	*rhaR*	Positive transcriptional regulator of L-rhamnose utilization.	Transcription
STM4119	4,336,890	T	G	Aspartate	Alanine	*ppc*	Phosphoenolpyruvate carboxylase, a cytosolic enzyme catalizing intermidiates of the citric acid cycle.	Metabolism
STM4257	4,478,065	A	G	Asparagine	Aspartate	STM4257	Hypothetical inner membrane protein.	Membrane
STM4258	4,478,688	T	C	Phenylalanine	Leucine	STM4258	Putative methyl-accepting chemotaxis protein.	Motility
STM4261	4,494,871	T	C	Tyrosine	Histidine	STM4261	Hypothetical inner membrane protein.	Membrane
STM4275	4,514,994	T	C	Aspartate	Glycine	*acs*	Acetyl-coenzyme A synthetase catalyzing intermediates at the junction of anabolic and catabolic pathways.	Metabolism
STM4549	4,807,787	C	G	Serine	Threonine	STM4549	Putative tRNA proofreading protein.	Metabolism
