# Supplementary material for: Prevalence and Genomic Diversity of Salmonella enterica Recovered from River Water in a Major Agricultural Region in Northwestern Mexico
Source: Microorganisms. 2022 Jun 14;10(6):1214. doi: 10.3390/microorganisms10061214 (PMC9228531; doi:10.3390/microorganisms10061214)
Supplement: Supplementary file 1 [file microorganisms-10-01214-s001.zip › Supplementary Table S3_Summary of Salmonella levels, water and environment parameters.pdf]

Supplementary Table S3. Summary of *Salmonella* levels, water and environment parameters for each sampling month.

	Salmonella [a]		Temperature (°C)		Rainfall (mm)		Relative Humidity (%)		Salinity (PSU)		Total Dissolved Solids (ppm)		Electrical Conductivity (μs/cm)		pH	
Date	Mean	CI [b] 95%	Mean	CI[b] 95%	Mean	CI[b] 95%	Mean	CI[b] 95%	Mean	CI[b] 95%	Mean	CI[b] 95%	Mean	CI[b] 95%	Mean	CI[b] 95%
Jun. 2018	13.9	(12.0, 15.8)	30.678	(29.3, 31.9)	32.91	(24.8, 40.9)	58.72	(57.81, 59.64)	1.005	(-0.58, 2.59)	259.2	(-984.6, 1503.0)	518	(-774, 1810)	7.59	(7.40, 7.78)
Jul. 2018	7.88	(5.98, 9.79)	30.057	(28.7, 31.3)	138.6	(130, 146)	64.00	(63.08, 64.91)	1.000	(-0.59, 2.59)	254.1	(-989.7, 1497.9)	508	(-784, 1800)	7.61	(7.42, 7.79)
Aug. 2018	6.75	(4.85, 8.66)	31.586	(30.3, 32.8)	164.9	(156, 172)	71.09	(70.17, 72.00)	1.005	(-0.58, 2.59)	391	(-853, 1635)	782	(-510, 2074)	7.57	(7.38, 7.76)
Dec. 2018	0.71	(-1.19, 2.6)	23.036	(21.7, 24.3)	21.27	(13.2, 29.3)	57.00	(56.08, 57.91)	0.962	(-0.62, 2.55)	429	(-815, 1673)	742	(-550, 2034)	7.35	(7.16, 7.54)
Jan. 2019	0.65	(-1.25, 2.5)	23.091	(21.8, 24.3)	15.54	(7.49, 23.5)	57.18	(56.26, 58.09)	1.112	(-0.47, 2.70)	853	(-391, 2097)	1049	(-243, 2341)	7.85	(7.66, 8.04)
Feb. 2019	0.73	(-1.17, 2.6)	23.427	(22.1, 24.7)	12.90	(4.85, 20.9)	57.54	(56.63, 58.45)	1.145	(-0.44, 2.73)	903	(-341, 2147)	1116	(-176, 2408)	7.80	(7.61, 7.98)
Mar. 2019	0.71	(-1.19, 2.6)	25.955	(24.6, 27.2)	4.727	(-3.3, 12.7)	52.00	(51.09, 52.91)	1.083	(-0.50, 2.67)	1411	(167, 2655)	1609	(317, 2901)	7.80	(7.61, 7.99)
Apr. 2019	1.21	(-0.69, 3.1)	26.009	(24.7, 27.2)	3.000	(-5.05, 11.0)	52.45	(51.54, 53.36)	1.169	(-0.42, 2.76)	1301	(57, 2545)	1552	(260, 2844)	7.86	(7.67, 8.05)
Jul. 2019	2.41	(0.51, 4.32)	32.845	(31.5, 34.1)	141.7	(133, 149)	63.45	(62.54, 64.37)	1.125	(-0.46, 2.71)	1250	(6, 2494)	1360	(68, 2652)	7.90	(7.71, 8.09)
Aug. 2019	2.33	(0.42, 4.23)	34.645	(33.3, 35.9)	162.7	(154, 170)	72.72	(71.81, 73.64)	1.030	(-0.56, 2.62)	1265	(22, 2509)	1339	(47, 2631)	7.88	(7.69, 8.07)
Sep. 2019	0.64	(-1.25, 2.5)	35.118	(33.8, 36.4)	186.6	(178, 194)	74.63	(73.72, 75.54)	1.056	(-0.53, 2.64)	1192	(-52, 2436)	1359	(67, 2651)	7.91	(7.72, 8.09)
Oct. 2019	0.87	(-1.03, 2.7)	25.336	(24.0, 26.6)	47.72	(39.6, 55.7)	71.63	(70.72, 72.54)	1.035	(-0.55, 2.62)	1139	(-105, 2383)	1275	(-17, 2567)	7.82	(7.63, 8.00)
Nov. 2019	0.93	(-0.96, 2.8)	24.636	(23.3, 25.9)	28.09	(20.0, 36.1)	66.27	(65.36, 67.18)	0.974	(-0.61, 2.56)	1056	(-187, 2300)	1165	(-127, 2457)	7.76	(7.57, 7.95)

[a] Estimation of *Salmonella* levels based on the most probable number (MPN) technique per 100 mL of sample [26].

[b] CI, confidence interval.
