# Supplementary material for: Prevalence and Genomic Diversity of Salmonella enterica Recovered from River Water in a Major Agricultural Region in Northwestern Mexico
Source: Microorganisms. 2022 Jun 14;10(6):1214. doi: 10.3390/microorganisms10061214 (PMC9228531; doi:10.3390/microorganisms10061214)
Supplement: Supplementary file 1 [file microorganisms-10-01214-s001.zip › Supplementary Tatble S4_Distribution of recovered Salmonella.pdf]

Supplementary Table S4. Distribution of recovered *Salmonella enterica* serovars by sampling site in the Culiacan Valley, Northwestern Mexico.

	**Number of recovered isolates of *Salmonella enterica* by site**												
	Humaya River			Tamazula River			Culiacan River						
Serovars	A	B	C	D	E	F	G	H	I	J	K	Total	Sites [b]
Oranienburg	1	2	2	2	1	2		1	4	1	1	17	10
Anatum		2	2		1		1					6	4
Saintpaul		3		1							1	5	3
Sandiego			1	1	1			1				4	4
Pomona								1	2	1		4	3
Give	2					1	1					4	3
Montevideo	2					2						4	2
Soahanina				1	1				1			3	3
Weltevreden		2						1				3	2
Braenderup					1		1					2	2
Bovismorbificans				1			1					2	2
Typhimurium					1			1				2	2
Javiana							1			1		2	2
Minnesota	2											2	1
Muenchen				1								1	1
Gaminara				1								1	1
Newport	1											1	1
Meleagridis							1					1	1
Albany			1									1	1
Abaetetuba					1							1	1
Agona							1					1	1
Seftenberg								1				1	1
Carrau											1	1	1
Saphra				1								1	1
Infantis						1						1	1
Poona						1						1	1
Yoruba			1									1	1
Total isolates recovered (Total distinct serovars) [a]	8 (5)	9 (4)	7 (5)	9 (8)	7 (7)	7 (5)	7 (7)	6 (6)	7 (3)	3 (3)	3 (3)	73 (27)	

[a] Number of *Salmonella* isolates serotypes detected by sample site

[b] Number of sites where each serotype was detected.
